# Therapeutic strategies for anchored kinases and phosphatases: exploiting short linear motifs and intrinsic disorder

**DOI:** 10.3389/fphar.2015.00158

**Published:** 2015-07-28

**Authors:** Patrick J. Nygren, John D. Scott

**Affiliations:** ^1^Department of Pharmacology, University of WashingtonSeattle, WA, USA; ^2^Howard Hughes Medical InstituteChevy Chase, MD, USA

**Keywords:** protein kinase A (PKA), protein phosphatase 2B (PP2B), calcineurin, A-kinase anchoring protein (AKAP), intrinsic disorder, cAMP signaling, short linear interaction motifs (SLiMs)

## Abstract

Phosphorylation events that occur in response to the second messenger cAMP are controlled spatially and temporally by protein kinase A (PKA) interacting with A-kinase anchoring proteins (AKAPs). Recent advances in understanding the structural basis for this interaction have reinforced the hypothesis that AKAPs create spatially constrained signaling microdomains. This has led to the realization that the PKA/AKAP interface is a potential drug target for modulating a plethora of cell-signaling events. Pharmacological disruption of kinase–AKAP interactions has previously been explored for disease treatment and remains an interesting area of research. However, disrupting or enhancing the association of phosphatases with AKAPs is a therapeutic concept of equal promise, particularly since they oppose the actions of many anchored kinases. Accordingly, numerous AKAPs bind phosphatases such as protein phosphatase 1 (PP1), calcineurin (PP2B), and PP2A. These multimodal signaling hubs are equally able to control the addition of phosphate groups onto target substrates, as well as the removal of these phosphate groups. In this review, we describe recent advances in structural analysis of kinase and phosphatase interactions with AKAPs, and suggest future possibilities for targeting these interactions for therapeutic benefit.

## Characterizing Protein Kinase A (PKA) Anchoring

The cAMP-dependent protein kinase A (PKA) was first identified and described by Edwin G. Krebs in 1968 as catalyzing the transfer of phosphate from ATP to a target serine or threonine residue in substrate proteins (Walsh et al., 1968). Since the initial identification of this ubiquitous kinase, many studies have defined its regulation by regulatory subunits (R-subunits), of which there are four isoforms (RIα, RIβ, RIIα, RIIβ; Taylor et al., 2012). PKA regulatory subunits inhibit the activity of the PKA catalytic subunit (C-subunit) by occupying the substrate binding site of the C-subunit and preventing the phosphorylation of substrate proteins (Corbin et al., 1978). When cAMP binds to the R-subunits and inhibition is released, the C-subunit is able to assume its catalytic activity and phosphorylate nearby targets. In addition, each R-subunit isotype contains an N-terminal docking and dimerization domain (D/D domain) that is the basis for the formation of a heterotetramer composed of two R-subunits, each of which bind one C-subunit (2:2 stoichiometry; Corbin et al., 1975; Newlon et al., 1999). In addition to the formation of R-subunit dimers, this D/D domain is responsible for docking to a genetically diverse but functionally related family of proteins called A-kinase anchoring proteins (AKAPs; Scott et al., 1990; Newlon et al., 2001).

The first AKAP to be identified was microtubule-associated protein 2 (MAP2) by analysis of associated cAMP-dependent kinase activity (Theurkauf and Vallee, 1982). The number of AKAPs identified since has vastly increased due to use of a far-western technique known as the RII overlay (Carr et al., 1991), as well as through more recent development of computational algorithms designed to predict R-subunit binding regions (Burgers et al., 2015). Some of the most characterized AKAPs include AKAP79/150, gravin, AKAP15/18, and mAKAP (Wong and Scott, 2004). In addition, some AKAPs have been shown to bind RI subunit isoforms, either with dual-specificity for RI and RII, or preference for the RI types (Huang et al., 1997a,b; Lacana et al., 2002; Kovanich et al., 2010; Means et al., 2011). However, the majority of AKAPs interact primarily with RII isoforms.

A-kinase anchoring proteins tether pools of readily stimulated PKA holoenzymes to subcellular compartments and organelles through a variety of mechanisms (Langeberg and Scott, 2015). Importantly, AKAPs also bind other signaling enzymes such as phosphodiesterases (PDEs), G-protein coupled receptors (GPCRs), ion channels, and protein phosphatases to form complexes that are able to integrate and modulate multiple second messenger signaling pathways and fine-tune cellular signaling responses. Many excellent reviews have described the range of binding partners these AKAPs associate with (Wong and Scott, 2004; Carnegie et al., 2009; Welch et al., 2010; Diviani et al., 2011; Sanderson and Dell’Acqua, 2011). In this review, we focus on the structural basis for anchoring of PKA as well as the protein phosphatases that oppose cAMP-mediated signaling.

## Structural Basis for PKA Anchoring

**FIGURE 1:**
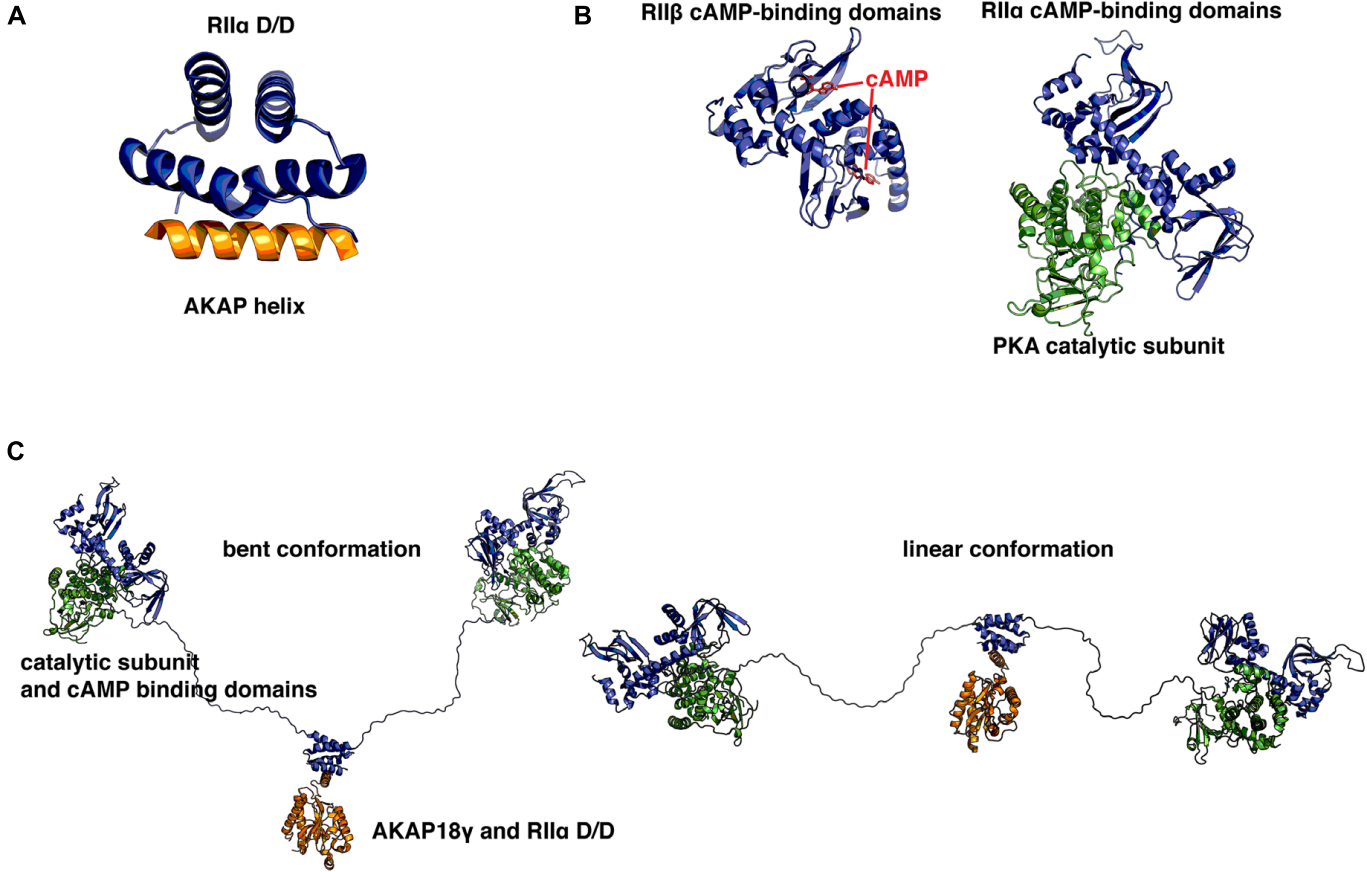
**Structural basis for protein kinase A (PKA) holoenzyme formation and anchoring. (A)** Crystal structure of the synthetic A-kinase anchoring protein (AKAP) helix AKAP’s (orange) in complex with the RIIα docking/dimerization (D/D) domain, residues 1–44 (blue). The AKAP amphipathic helix binds to a hydrophobic groove created by the antiparallel X-type helix bundle of the RII D/D domain. PDB ID: 2IZX. **(B)** Left: RIIβ cAMP-binding cassettes (blue) in complex with cAMP (red). Right: RIIα cAMP binding cassettes in complex with PKA catalytic subunit (green). With cAMP bound at each of two sites, RII releases inhibition of the catalytic subunit. When cAMP is not present, RII presents an inhibitory sequence to the active site, preventing phosphorylation of PKA substrates. PDB IDs: 1CX4, 2WVS. **(C)** A pseudo-atomic model of the PKA holoenzyme in complex with AKAP18γ derived from low-resolution EM data. This illustrates that the PKA holoenzyme has a constrained range of flexibility (∼300 Å) provided by AKAPs, allowing the catalytic subunits to be poised near potential substrates. PDB IDs: 3J4Q, 3J4R. Models were prepared using PyMol (Schrödinger).

Though AKAPs are not typically related to one another on a sequence level, a common unifying feature is their ability to bind the D/D domain of R-subunit dimers through a short (14–18 residues) amphipathic helix, which appears to have arisen relatively early in evolution (Peng et al., 2015). This helix is often one of the few ordered regions, as most AKAPs are intrinsically disordered (Gold et al., 2008). Therefore, this helix serves as a short linear motif (SLiM), which is an emerging concept in cellular signaling that has important implications for protein–protein interactions and drug development (Van Roey et al., 2014). For example, a recent study examining the scaffolding properties of the yeast deubiquitinating enzyme Ubp10 showed that the interplay of SLiMs and intrinsic disorder is essential for facilitating interactions with diverse substrates and binding partners (Reed et al., 2015). SLiMs are often isolated within intrinsically disordered proteins and can serve to facilitate transient interactions which allows a single anchoring protein to interact with a dynamic range of signaling partners (Ren et al., 2008). The first atomic model of an AKAP helix was solved using peptides derived from AKAP79 and Ht31 (AKAP-Lbc) (Newlon et al., 1999). It was obtained using NMR techniques and was solved in complex with the D/D domain (residues 1–44) of RIIα. Subsequently, other structures of the D/D domain in complex with various AKAP-derived helices have been solved using X-ray crystallography (**Figure 1A**; Gold et al., 2006; Kinderman et al., 2006; Sarma et al., 2010). The D/D domain has been shown to adopt an anti-parallel four-helix X-type bundle that forms a platform with a hydrophobic groove. This groove is the basis for a high affinity interaction with the hydrophobic face of amphipathic AKAP helices. The D/D domain is then connected via a flexible linker to two cAMP-binding cassettes per protomer that display cooperative binding of cAMP (Vigil et al., 2004; Zawadzki and Taylor, 2004). Upon binding of cAMP, a conformational change occurs that relieves inhibition of the PKA C-subunit and allows it to phosphorylate nearby substrates. Crystal structures have been solved for the cAMP-binding cassettes in complex with C-subunit or with cAMP (**Figure 1B**; Su et al., 1995; Diller et al., 2001; Wu et al., 2007; Zhang et al., 2012). Together with the known structure of the D/D in complex with AKAP helices, these structures have provided insights at the atomic level about the intricate topology and organization of the different functional elements of PKA holoenzyme.

Yet, there is currently no high-resolution structural information available for the 46 (in mammals) amino acid flexible linker that connects the D/D domain to the pseudosubstrate region that binds the C-subunit and to the tandem cAMP binding cassettes. Therefore, a recent study used single particle electron microscopy studies to examine the structure of an AKAP18γ-PKA holoenzyme complex (Smith et al., 2013). This study revealed that although many crystal structures of RII and C-subunits showed surface contact between each heterodimer of RII and C, the complexes likely occupy a much broader conformational space that is constrained by the length of the linker, yet facilitated by the intrinsic disorder of the linker (**Figure 1C**). This linker-guided conformation sampling may be a mechanism by which PKA preferentially phosphorylates substrates within the same macromolecular complexes upon elevation of cAMP levels. cAMP PDEs have been suggested to form a ‘fence’ around subcellular pools of elevated cAMP (Baillie et al., 2005). AKAP18γ has been shown to form a complex with PDE4D3 and regulate its activity via PKA phosphorylation (Stefan et al., 2007). In combination with local restraint of PKA conformations by the RII flexible linker, these local PDE fences represent an intriguing scheme by which spatiotemporal specificity may be regulated by macromolecular signaling complexes.

## Targeting the PKA/AKAP Interaction for Therapeutics

Since PKA activity modulates a variety of physiological events, such as cardiac remodeling, disrupting the PKA/AKAP interface has been a long-standing area of interest for therapeutics (Troger et al., 2012; **Table 1**). One of the first disruptors of the AKAP/RII interaction is the 24 amino acid peptide Ht31, named after human thyroid clone 31, which was later realized to represent a biologically active segment of the multifunctional scaffolding protein AKAP-Lbc (Carr et al., 1992). The Ht31 peptide has since been lipid modified with a stearol group to increase its membrane permeability for treatment of cell lines and elucidation of anchored PKA signaling events (Vijayaraghavan et al., 1997; Gold et al., 2012). *In silico* approaches have resulted in optimized peptides that mimic the AKAP amphipathic helix and bind to RII or RI with high affinity (Alto et al., 2003). Added to this, structure-based approaches have further increased the specificity of the peptide superAKAPis for RII to the low nanomolar affinity range with a 12,000-fold preference for RII over RI (Gold et al., 2006). Conversely, the RI-anchoring disruptor peptide (RIAD) has been engineered to specifically disrupt RI/AKAP interactions (Carlson et al., 2006). Peptidomimetics have been developed by several groups that mimic amphipathic helix structures and are able to disrupt RI/RII interactions with AKAPs (Schafer et al., 2013; Singh et al., 2014). Recent work has centered on developing stapled AKAP-mimetic peptides that are cell-permeable and have increased stability (Wang et al., 2014, 2015; Kennedy and Scott, 2015). This would increase the utility of these peptides for therapeutic purposes as well as for teasing apart the molecular mechanisms by which AKAPs influence local PKA signaling pathways.

**Table 1:** Summary of molecules disrupting protein kinase A (PKA) anchoring.

Name	Type/mechanism	Reference
Ht31	Derived from A-kinase anchoring protein (AKAP) helix	Stokka et al. (2006)
SuperAKAPis	Optimized AKAP helix	Gold et al. (2006)
RI-anchoring disruptor peptide (RIAD)	Optimized AKAP helix	Carlson et al. (2006)
STAD peptides	Stapled AKAP helix	Wang et al. (2014, 2015)
RIAD-P3	Peptidomimetic of RIAD	Singh et al. (2014)
Terpyridine derivatives	Peptidomimetic of AKAP helix	Schafer et al. (2013)
Rselects	Engineered R-subunit D/D domain	Gold et al. (2013)
FMP-API-1	Allosteric interaction with R-subunits	Christian et al. (2011)

Since there are numerous AKAPs and only four R-subunit isoforms, any disruptor that relies on an interaction with an R-subunit is by definition non-selective. In order to disrupt a specific AKAP’s ability to bind PKA, anchoring disruptors must bind to an AKAP helix with high affinity and recognize the unique structural features of one AKAP helix preferentially. Therefore, a phage-display screening approach, which used immobilized AKAP helices to enrich for phage variants that displayed mutant RII D/D domains, selected variants that exhibit preferential binding to specific AKAPs. These mutant D/D domains are termed Rselects, and have been shown in preliminary work to bind and label AKAPs in a cellular context as well as in to purified proteins (Gold et al., 2013). Further development of these Rselects could lead to high affinity binding variants that could disrupt individual pools of anchored PKA while allowing other anchored PKA signaling events to proceed unperturbed. The potential to isolate spatially constrained post-translational modifications is an important step forward for targeted therapeutics. However, utility of this approach as a cell based means of selectively interrupting particular PKA–AKAP interfaces has yet to be rigorously established.

Small molecule disruptors are another attractive means to pharmacologically target the PKA–AKAP interface. Although these studies are still in their formative stages there have been a few successful attempts at moderate-throughput screening for small molecule AKAP disruptors (Schachterle et al., 2015). Perhaps the most notable example is the development of 3,3′-diamino-4,4′-dihydroxydiphenylmethane (FMP-API-1), a small molecule antagonist that appears to allosterically inhibit the RII–AKAP interaction and activate anchored PKA C-subunit (Christian et al., 2011). Yet, despite extensive characterization of this compound the mechanism of action of FMP-API-1 has yet to be defined. Nonetheless, the future is bright for the discovery and development of cell soluble chemical entities that target PKA–AKAP interfaces.

## Protein Phosphatase Anchoring

Classically, protein phosphatases are considered to be responsible for the opposing action to kinases, namely the removal of phosphate groups from serine, threonine, or tyrosine residues. In addition a burgeoning family of pseudokinases and pseudophosphatases are emerging a key players in cell signaling (Reiterer et al., 2014). Protein phosphatases fall into two main classes – serine–threonine phosphatases, and tyrosine phosphatases. While there are 428 serine/threonine kinases, there are only ∼40 serine/threonine phosphatases (Moorhead et al., 2007). This disparity in gene number infers that additional mechanisms come into play as a means to modulate and vary the substrate specificity of these critical regulatory enzymes. Philip and Tricia Cohen were the first to recognize that regulation of protein phosphatases by association with regulatory and targeting subunits is a crucial mechanism to allosterically modulate substrate specificity (Stralfors et al., 1985; Cohen and Cohen, 1989). Subsequently others have shown that most of the three classes of serine/threonine phosphatases are modulated by targeting subunits (Langeberg and Scott, 2015). In this article we focus exclusively on protein phosphatase 1 (PP1) and protein phosphatase 2B (PP2B, or calcineurin), since these ubiquitous phosphatases often oppose the action of PKA and are especially reliant on anchoring for their regulation.

## Protein Phosphatase 1 Regulation by Auxiliary Proteins

Protein phosphatase 1 has an important role in a number of physiological processes, notably regulation of glycogen synthesis (Hubbard et al., 1990), nuclear events (Helps et al., 1998), and synaptic long term potentiation (LTP) and long term depression (LTD), (Morishita et al., 2001; Malinow and Malenka, 2002). The latter two events occur through phosphatase opposition of CaMKII and PKA phosphorylation of glutamate receptors at the post-synaptic density. The PP1 catalytic subunit (PP1c) associates with over 200 regulatory subunits, many of which bind via a conserved short linear peptide motif called the RVxF motif (Cohen, 2002; Roy and Cyert, 2009).

**FIGURE 2:**
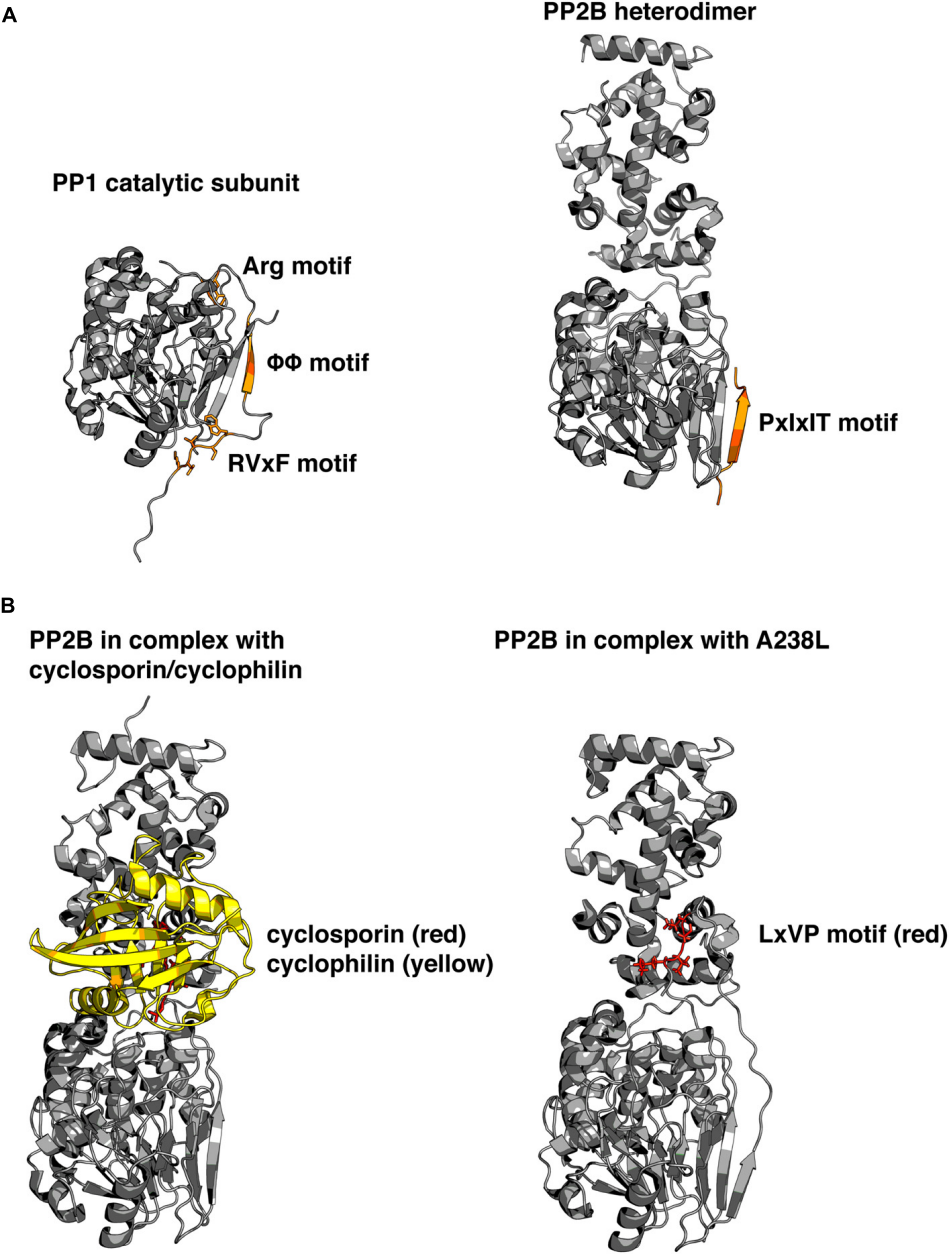
**Structural basis for phosphatase regulation and anchoring. (A)** Left: PP1 catalytic subunit (gray) in complex with RVxF and auxiliary anchoring motifs from protein phosphatase 1 (PP1) nuclear targeting subunit (PNUTS; orange). Right: PP2B (gray) in complex with PIAIIIT sequence from AKAP79 (orange). Comparison reveals that similar surfaces are used for anchoring, and that multiple motifs can simultaneously interact with varied portions of the molecule. PDB IDs: 4MOY, 3LL8. **(B)** Left: PP2B in complex with cyclosporin (red)/cyclophilin (yellow) complex. Right: PP2B in complex with a viral peptide A238L, containing a PxIxIT motif, as well as an LxVP motif (red). Cyclosporin and LxVP peptides bind to overlapping surfaces on PP2B, formed by both the catalytic and regulatory subunits of PP2B. This surface does not occlude the active site of the phosphatase, yet immunosuppressants are able to allosterically inhibit PP2B activity toward substrates. PDB IDs: 1MF8, 4F0Z. Models were prepared using PyMol (Schrödinger).

Some of these subunits serve primarily to inhibit the catalytic activity, such as the protein Inhibitor 1 (I-1) and dopamine and cAMP-regulated phosphoprotein 32 (DARPP32), (Williams et al., 1986). Notably some of these inhibitors are activated by PKA phosphorylation. Other regulatory subunits contain localization signatures that target PP1 to specific subcellular regions and may or may not also inhibit the enzymatic activity of PP1c. The most recognized examples of these targeting subunits are the myosin phosphatase targeting subunit MYPT1, the G_M_ regulatory subunit, p53-binding protein 2 (53BP2), and PP1 nuclear targeting subunit (PNUTS). Recent investigation of PNUTS has highlighted several properties shared by many PP1-binding proteins (Choy et al., 2014). First, the RVxF motif serves as a short linear interaction motif (SLiM) and is responsible for the primary interaction. Second, intrinsic disorder in PNUTS facilitates extended contact with PP1 on additional surfaces to fine-tune the phosphatase. Third, binding to these surfaces inhibits activity toward some substrates without physically blocking the active site of the phosphatase (**Figure 2A**).

A number of AKAPs have been shown to interact with PP1c, including AKAP220 (Schillace and Scott, 1999), D-AKAP1 (Steen et al., 2000), and yotiao (Westphal et al., 1999). Likewise, some isoforms of AKAP18 are thought to sequester PP1, although it would appear that this occurs via indirect mechanisms (Singh et al., 2011). All direct PP1–AKAP interfaces utilize some version of the degenerate RVxF motif. D-AKAP1 was suggested to be involved in anchoring PP1 for efficient nuclear envelope reassembly after mitosis (Collas et al., 1999; Steen et al., 2000). AKAP220 has been shown to anchor PP1 through a modified KVxF motif, and this has been proposed to play a role in regulating the activity of glycogen synthase kinase 3β (GSK3β), through modulation of the phosphorylation state of serine 9. Phosphorylation of this residue results in suppression of GSK3β activity (Schillace et al., 2001; Tanji et al., 2002; Whiting et al., 2015). Yotiao, a product of the AKAP9 gene, also contains an RVxF motif, and has been shown to regulate the phosphorylation state of NMDA receptors through localization of PP1 (Lin et al., 1998; Westphal et al., 1999). AKAP18 does not appear to interact directly with PP1, but some reports indicate that it binds Inhibitor-1 to promote its phosphorylation by PKA (Singh et al., 2011). The net effect of this later phosphorylation event is to promote local inhibition of PP1c.

## PP2B Regulation by Auxiliary Proteins

PP2B, also known as calcineurin, is a broadly expressed obligate heterodimeric protein phosphatase that is activated by calcium and calmodulin (Stewart et al., 1982). Like PP1, PP2B is involved in diverse processes such as synaptic plasticity (Mulkey et al., 1994), glucose metabolism (Hinke et al., 2012), cardiac signaling (Tandan et al., 2009), and immune responses (Clipstone and Crabtree, 1992). In addition, activation of PP2B can mobilize phosphatase cascade, through dephosphorylation of PP1 regulatory subunits (Mulkey et al., 1994). The catalytic A subunit of PP2B contains an autoinhibitory region that occludes the active site in the absence of calcium. Upon elevation of calcium levels, calcium ions bind directly to the regulatory B subunit, and to calmodulin, which in turn interacts with the autoinhibitory region and allows PP2B to resume catalytic activity toward phosphosubstrates (Li et al., 2011). Because calcium transients often envelop the whole cell rather than occurring locally, regulation of PP2B’s activity toward substrates is accomplished primarily through protein–protein interactions. The best-known PP2B substrate is the nuclear factor of activated T-cells (NFAT) family. These transcription factors contain phosphoserine-rich regions, and when dephosphorylated, dimerize and translocate to the nucleus, where they are responsible for controlling a range of transcriptional responses such as inflammation in response to immune system signaling (Li et al., 2012). The common immunosuppressants FK506 and cyclosporine target PP2B and have their primary effect through inhibition of NFAT signaling (Liu et al., 1991).

Not only is NFAT a typical PP2B substrate, it also contains two SLiMs, which are typical of PP2B interacting proteins – the PxIxIT motif, and the LxVP motif (Roy et al., 2007; Rodriguez et al., 2009). The PxIxIT motif forms a beta strand that binds to a hydrophobic groove formed by a beta sheet on the PP2B A subunit (Li et al., 2007). This surface of the PP2B A subunit is analogous to the region of PP1 which interacts with the RVxF motif (**Figure 2A**). Proteins that contain PxIxIT motifs include NFAT, regulator of calcineurin 1 (RCAN1; Mehta et al., 2009), TWIK-related spinal cord potassium channel (TRESK; Roy and Cyert, 2009), and notably, AKAP79/150 (Dell’Acqua et al., 2002). The LxVP motif is a degenerate sequence that binds to the interface of the A and B subunits of PP2B, and only binds to activated calcineurin (Rodriguez et al., 2009). It has been challenging to describe a consensus LxVP sequence. Therefore, many LxVP motifs have been identified without originally being aware of their identity. The first LxVP motif to be described was that of the RII subunit by Blumenthal et al. (1986), although it was not recognized as a conserved binding mode until it was found in NFAT. Many substrates of PP2B contain an LxVP motif, and it has been suggested that all efficient substrates contain some type of sequence that interacts with the LxVP binding region on PP2B (Grigoriu et al., 2013). The characterization of multiple SLiMs that interact with various surfaces of PP2B parallels that of PP1, and suggests that other mechanisms may also be in common such as fine-tuning the location and activity of PP2B through a combination of disorder and SLiMs. Recently, a structure of PP2B in complex with a viral inhibitor peptide from African swine fever revealed the binding site for the LxVP motif in atomic level detail (Grigoriu et al., 2013). This crystal structure reveals that the leucine residue occupies a pocket formed by two aromatic residues, and when these are mutated to alanine residues they no longer interact with the LxVP motif. In addition, this binding site overlaps with the binding sites for cyclosporine and FK506 complexes (**Figure 2B**). However, no structure has been solved of the PP2B heterodimer bound to calmodulin in the fully active state, so the question of how LxVP motifs are able to impact PP2B activity remains unclear.

Some AKAPs have been shown to bind PP2B, such as the aforementioned AKAP79, and mAKAP (Li et al., 2010). In addition, the AKAP gravin has been suggested to be in the same complex as PP2B and beta-adrenergic receptors, however, evidence for a direct interaction is not immediately apparent (Shih et al., 1999). The mAKAP interaction has been mapped to the residues 1286–1345 in the mAKAPα splice variant. However, this region does not contain an easily identifiable PxIxIT or LxVP sequence. Loss of mAKAP-PP2B binding was shown to result in reduced cardiac myocyte hypertrophy in response to norepinephrine, as well decreased atrial natriuretic factor expression. Interestingly, the pool of PP2B bound to mAKAP appeared to be active, and required to dephosphorylate NFAT efficiently in response to phenylephrine treatment. Formation of the PP2B/mAKAP complex was enhanced *in vitro* by calcium/calmodulin, suggesting that the interaction may occur via a similar mechanism to the LxVP motif (Li et al., 2010).

A-kinase anchoring protein 79 is perhaps the best characterized AKAP, and its interaction with PP2B has been extensively investigated. Although original studies suggested an interaction site was restricted to the N-terminal third of AKAP79 (Coghlan et al., 1995), later work described the primary site of interaction as being a PxIxIT motif from residues 337-343 (Dell’Acqua et al., 2002; Oliveria et al., 2007). Use of a transgenic mouse model in which AKAP79 lacks this region, known as the AKAP79ΔPIX mouse, has revealed that AKAP79-anchored PP2B is required for NMDA-dependent hippocampal long-term depression and NFAT signaling in neurons (Oliveria et al., 2007; Sanderson et al., 2012). Intriguingly, the AKAP79ΔPIX mouse shows improved insulin sensitivity, indicating that this interaction may be a possible therapeutic target for Type II diabetes (Hinke et al., 2012).

Because of the importance of the AKAP79–PP2B interaction, much emphasis has been placed on understanding the structural basis of this interaction. Native mass spectrometry and biochemical approaches have suggested that there is an additional interaction site for PP2B between residues 1–153 of AKAP79 that is dependent on calcium/calmodulin (Gold et al., 2011). Although a crystal structure of PP2B in complex with a synthetic PxIxIT motif was solved in 2007 (Li et al., 2007), the first structure of PP2B bound to a natural PxIxIT motif was that of AKAP79 (Li et al., 2012). This structure matched closely with the previously solved structure, in that crystal packing is such that each PIAIIIT sequence contacts two PP2B A subunits along the PxIxIT binding region. This, along with native mass spectrometry approaches, raises the question of whether AKAP79 is capable of binding two PP2B molecules simultaneously.

Because of PP2B’s importance in a range of physiological contexts, there is great interest in developing disruptors that target specific PP2B anchoring proteins (**Table 2**). One of the first targeted approaches resulted in an optimized high-affinity PxIxIT motif called the VIVIT peptide (Aramburu et al., 1999), which is the aforementioned synthetic peptide that was co-crystallized with PP2B. In addition, fluorescence polarization screens for small molecules that disrupt binding to the PxIxIT motif have yielded a potential candidate known as INCA-6 that is able to inhibit PP2B-NFAT signaling with similar potency to cyclosporine and FK506, but through an alternate mechanism (Kang et al., 2005). Recently, an approach disrupting the LxVP interaction in macrophages through lentiviral expression of an LxVP peptide was shown to reduce inflammation and confer resistance to arthritis and contact hypersensitivity (Escolano et al., 2014). Understanding the molecular basis for PP2B anchoring has led to potential for therapeutics and the realization that primary and secondary binding sites may both be targeted for diverse physiological effect. Because the AKAP79–PP2B interaction is important in many processes, specifically targeting this interaction may be of great promise.

**Table 2:** Summary of molecules disrupting protein phosphatase-2B (PP2B) anchoring.

Name	Type/mechanism	Reference
Cyclosporine	Immunophilin complex, competes with LxVP	Liu et al. (1991)
FK506	Immunophilin complex, competes with LxVP	Liu et al. (1991)
VIVIT	Optimized PxIxIT peptide	Aramburu et al. (1999)
INCA-6	Allosteric disruptor of PxIxIT	Kang et al. (2005)
LxVP peptide	LxVP peptide derived from NFAT	Escolano et al. (2014)
PxIxIT disruptors from ZINC library	Organic compounds, direct competition for PxIxIT	Matsoukas et al. (2015)

## Conclusion

Protein kinase A phosphorylation events and the phosphatases that oppose them are tightly regulated by anchoring proteins. Recently, the use of new and sophisticated biochemical, biophysical, and structural techniques have forged two important concepts. First, the combination of SLiMs and intrinsic disorder allow anchoring proteins to allosterically and spatially control the range and specificity of phospho-signaling. Second, AKAPs are not just static anchors, but are conformationally and compositionally flexible. This allows them to adapt to a varied and continually changing cellular signaling environment. A recent paper characterizing binding partners of the AKAP ezrin by quantitative mass spectrometry revealed that conformational switches in ezrin are accompanied by changes in the complement of enzymes present in the complex (Viswanatha et al., 2013). This may well prove to be the case for many AKAPs allowing them to perform cell type specific roles. Moreover, the concept of AKAPs as conformational switches could account for how the same anchoring protein can simultaneously perform distinct functions at multiple locations within a single cell.

These new biological insights have been demonstrated by using hybrid structural techniques such as x-ray crystallography, NMR, hydrogen/deuterium exchange experiments and crosslinking/mass spectrometry (Burns-Hamuro et al., 2005; Gold et al., 2011; Choy et al., 2014). The advent of direct electron detectors for cryo-electron microscopy has increased attainable resolutions (Campbell et al., 2012), and will likely contribute to increased structural understanding of these flexible multi-protein complexes. In addition, computational advances in understanding heterogeneous cryo-EM samples will also advance our knowledge of multiple conformational states (Behrmann et al., 2015). Already, negative-stain approaches such as random conical tilt (RCT) experiments are allowing researchers to understand structural heterogeneity in protein complexes (Veesler et al., 2014). Combining these approaches with biosensors for enzymatic activity (Mehta and Zhang, 2014; Mehta et al., 2014) will provide a more comprehensive picture of how the structural properties of anchored kinase and phosphatase complexes are able to influence local signaling in a cellular context. Finally, as exemplified by a recent structure-guided pharmacophore screen for inhibitors of PP2B anchoring, atomic resolution structural insights will guide design of small molecules that target anchoring protein interactions in the context of SLiMs and intrinsic disorder (Matsoukas et al., 2015).

## Author Contributions

PN, JS: Wrote, edited, and approved final version of the manuscript and figures.

## Conflict of Interest Statement

The authors declare that the research was conducted in the absence of any commercial or financial relationships that could be construed as a potential conflict of interest.
